# Technology-Assisted Neuromotor Training for Improving Visuomotor Reaction Performance, Change-of-Direction Quickness, and Bilateral Task Execution in Junior Handball Players

**DOI:** 10.3390/jfmk11010042

**Published:** 2026-01-20

**Authors:** Mircea Boncuț, Nicola Mancini, Angel-Alex Hăisan, Delia Boncuț, Emilia Florina Grosu, Cornelia Popovici, Carlos Hervás-Gómez, Cristina Maria Man, Siria Mancini, Mariasole Antonietta Guerriero, Antonella De Maria, Vlad Teodor Grosu

**Affiliations:** 1 Faculty of Physical Education and Sport, “Babes-Bolyai” University, Mihail Kogălniceanu Street 1, 400347 Cluj-Napoca, Romania; mircea.boncut@ubbcluj.ro (M.B.); delia.boncut@ubbcluj.ro (D.B.); emilia.grosu@ubbcluj.ro (E.F.G.); 2 Department of Science of Education and Sport, Pegaso Telematic University, 80143 Naples, Italy; 3 Department of Physical Education and Sport, “1 Decembrie 1918” University of Alba Iulia, Gabriel Bethlen Street 5, 510009 Alba Iulia, Romania; cristina.man@uab.ro; 4 Physical Education Discipline, University of Medicine and Pharmacy “Iuliu Hațieganu”, Pasteur Street 6, 400347 Cluj-Napoca, Romania; cornelia.popovici@umfcluj.ro; 5 Department of Teaching and Educational Organization, University of Sevilla, 41013 Sevilla, Spain; hervas@us.es; 6 Department of Humanistic Studies, University of Foggia, 71121 Foggia, Italy; siria_mancini.585707@unifg.it (S.M.); mariasole.guerriero@unifg.it (M.A.G.); 7 Section of Human Physiology, Unit of Dietetics and Sports Medicine, Department of Experimental Medicine, Università degli Studi della Campania “Luigi Vanvitelli”, 80138 Naples, Italy; antonella.demaria@unipegaso.it; 8 Faculty of Industrial Engineering, Robotics, and Production Management, Technical University of Cluj-Napoca, Muncii Street 103-105, 400461 Cluj-Napoca, Romania; vlad.grosu@mdm.utcluj.ro

**Keywords:** reaction time, visuomotor coordination, handball, youth sports, BlazePod, movement quickness, change-of-direction performance, visual stimuli

## Abstract

**Background:** Reaction time and coordination are key performance components in team sports such as handball, particularly during the developmental years. Integrating visual and cognitive stimuli through smart technologies has been shown to facilitate motor skill development in young athletes. **Methods:** This study evaluated the effects of a BlazePod-based training protocol on reaction time, visuomotor coordination, movement quickness, and change-of-direction performance in junior male handball players aged 12–14 years. Thirty-two athletes (mean age = 13.37 ± 0.29 years) were randomly assigned to an experimental group (n = 16), in which the traditional neuromotor/coordination block of regular practice was replaced with BlazePod-based drills three times per week for eight weeks, or to a control group (n = 16), which trained the same capacities with traditional handball-specific exercises without technology. Training frequency (3 sessions/week), session duration (90 min), and the workload of the 30 min neuromotor block were matched between groups. Motor performance was assessed using four tests: Focus Reactions, Fast Feet, Clap Challenge, and the Agility T-Test. Paired- and independent-samples *t*-tests were applied to compare pre- and post-intervention scores. **Results:** The experimental group showed significant within-group improvements in Focus Reactions (*p* = 0.002) and AgilTT_ShuffleLeft (*p* = 0.014), whereas the control group showed no improvements and a small but significant worsening in Focus Reactions. Between-group comparisons at post-test revealed significant differences in favor of the experimental group for Fast Feet (*p* = 0.036), Clap Challenge (*p* = 0.008), AgilTT_Overall (*p* < 0.001), and AgilTT_SprintBack (*p* = 0.003). **Conclusions:** The integration of BlazePod technology into handball training produced measurable improvements in reaction speed and lateral agility among junior players. These findings suggest that technology-assisted neuromotor training represents a viable training modality that can replace a traditional neuromotor block within youth handball practice while maintaining overall training dose.

## 1. Introduction

Handball is a complex sport with an accelerated training dynamics based on factors of advancement and improvement, some of which are novelties, while others are updates and adaptations to higher levels. It is a team sport that necessitates the application of critical physiological, psychological, and motor performance variables [1]. In team sports, the significance of reacting swiftly to stimuli, paired with high levels of synchronization, provides a firm foundation for success in competitive circumstances [2].

Handball places intense physical and cognitive demands on players, involving technical–tactical skills, reaction speed, coordination, and adaptability in dynamic game contexts [3,4]. Junior players are at a key stage of motor and cognitive development, which calls for age-appropriate modern training methods [5,6]. Recent evidence shows that performance depends not only on strength and endurance but also on coordination, agility, reaction time, and the capacity to process information in real time [7,8,9].

Between ages 10–13, coordination develops rapidly, supporting the acquisition of technical–tactical elements. Emphasis on coordination at this age leverages the nervous system’s plasticity to refine and correct motor habits [10]. Improving coordination is directly linked to maximizing technical–tactical potential and efficiency under stress [11,12]. Lower-limb asymmetries may negatively affect agility and explosive strength [13], while laterality and dexterity influence execution and decision-making [14,15,16,17]. Targeted plyometric exercises [18,19,20,21,22] and speed–agility–quickness training [23] improve youth performance.

The sensory–motor component is crucial to handball success and should be addressed with programs tailored to age and player characteristics [24]. Training that challenges visual, cognitive, and oculomotor perception can enhance processing skills and athletic performance [25]. Players and coaches therefore seek effective, modern instructional methods [26]. Anthropometric traits relate to movement and execution speed and can be used to benchmark performance [27].

Purposeful development of movement quickness and change-of-direction abilities, through action-based and isometric work, should be integrated into handball-specific training routines [28].

Motor skills are highly adaptable and expressed through posture, attitudes, and movements in ever-changing environments [29,30]. Psychomotor abilities optimally develop up to ~14 years, provided activities are structured and scientifically guided [31]. Technology is increasingly used to train and assess visuomotor reaction, manuality/laterality, and eye–hand/eye–foot coordination in performance sport [32].

Customized programs may include small-sided games and focused themes using modern interactive technologies [33].

Digital training tools (e.g., light-based systems) have gained popularity for improving reaction time and decision-making in interactive settings [34,35].

These technologies enable the integration of visual, auditory, and motor instruction while also triggering neuromuscular and perceptual responses [36,37,38,39]. Furthermore, research in the field of enhancing visual and auditory reaction time [40,41,42] demonstrates that these abilities can be improved by specific workouts, particularly in the context of dual tasks [43] or the integration of peripheral perception [35].

Young athletes who use technology to perform age-appropriate training have shown an improvement in response time and hand-eye and foot-eye coordination [44]. Functional asymmetry in the cerebral hemispheres is determined by the motor superiority of the manual or foot laterality, which categorizes patients as right-handed or left-handed, ambidextrous, or having crossed laterality [45]. The employment of BlazePod technology for cognitive enrichment in training can improve young athletes’ motor performance [46].

A further essential element is the preservation of balance and postural control, which are important components of coordination [47,48]. In the case of handball, these have a direct impact on technical execution and adaptability during duels [49]. Furthermore, motivating intents and exercise implementation planning differ with age [50], which is an important consideration when selecting and adapting training programs for juniors.

Given these demands, integrating a light-based reaction training system into handball practice may improve speed and coordination. This study evaluates its impact on junior players’ speed and coordination, building on the theoretical and empirical bases summarized above.

## 2. Materials and Methods

This study was designed to evaluate the effectiveness of a light-based sensor system (BlazePod^TM^; PlayCoy Ltd., Tel Aviv, Israel) in performance sport settings to improve reaction time, visuomotor coordination, movement quickness, agility change-of-direction performance, and bilateral task execution. Session plans were standardized a priori and implemented by the coaching staff to ensure comparable exposure time and bout structure across groups. Both groups completed the same training frequency (3 sessions/week) and session duration (90 min) over the 8-week intervention. In both groups, a 30 min neuromotor block at the beginning of each session targeted visuomotor reaction, change-of-direction quickness, and bilateral task execution; the workload of this block (bout duration, recovery duration, repetitions, and total time-on-task) was matched between groups. The only difference was the training modality: BlazePod-based drills (EG) versus traditional handball-specific drills without electronic devices (CG). The intervention took place between October 2024 and February 2025, covering both pre-season and in-season periods. The experimental group trained at the Ana Aslan Technical College gym in Cluj-Napoca, while the control group trained at the Gheorghe Barițiu gym in Turda. Informed consent was obtained from all participants and their legal guardians. Consent was also approved by the management of both sports clubs.

### 2.1. Participants

Thirty-two male junior handball players (National Junior Championship 3, FRH), aged 12–14, with ≥2 years of competitive experience, valid medical certificates, and regular team training participation, volunteered. Exclusion criteria: health issues, above-average BMI, recent injury, or ongoing rehabilitation.

Group allocation was performed using a simple randomization procedure. After eligibility assessment, participants were randomly assigned to either the Experimental Group (EG) or the Control Group (CG) using a random allocation sequence generated by an independent researcher not involved in data collection or training delivery. Group assignment was conducted prior to the start of the intervention, and all participants completed the study according to their allocated group.

### 2.2. Ethics and Data Protection

All participants were informed about the objectives, procedures, and their rights before providing consent. Personal data and test results were processed in accordance with GDPR (EU 2016/679), ensuring confidentiality and full anonymization. The study was approved by the Institutional Ethics Committee of Pegaso Telematic University (PROT/E 002466, 29 March 2024) prior to data collection. All procedures adhered to institutional and national guidelines for human-subjects research.

### 2.3. Equipment and Tests

**Figure 1:**
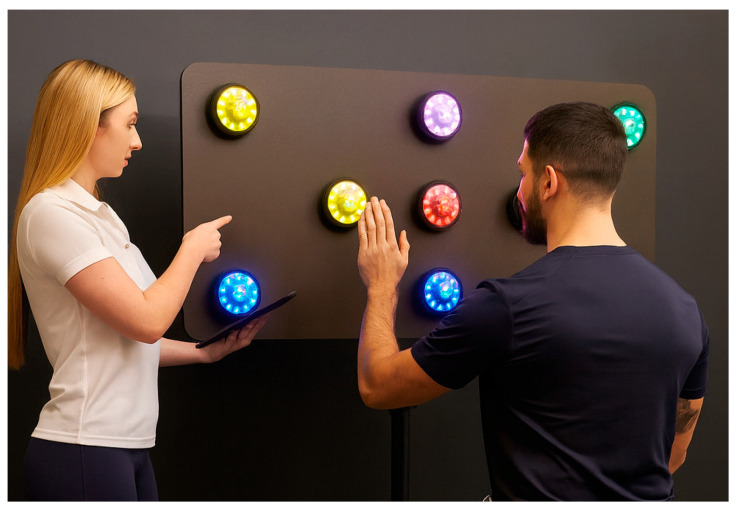
Example setup of a light-based sensor system.

As a light-based sensor system (Figure 1), this technology has received limited attention in performance research. It is primarily a measurement and feedback tool, but recent evidence suggests that it may also support neuromotor training to improve performance, reaction time, and perceptual–cognitive skills [34].

The following tests were administered; the best performance within the allotted window was recorded:

- Focus Reactions Test—reaction time, decision-making, cortical activity (neuronal firing).
- Fast Feet Test—decision-making, reaction speed, neuronal activity.
- Clap Challenge Test—hand–eye coordination and reaction speed.
- Agility T Test (AgilTT)—multidirectional acceleration and deceleration, change-of-direction (COD) performance, and movement quickness.
  - Anthropometry: height (cm) and body mass (kg) were measured with a Momert 5968 digital scale with a stadiometer (Momert Ltd., Subotica, Serbia); participants wore shoes and removed bulky clothing.

Outcome measures were collected only during standardized pre- and post-test sessions (M1 and M2), under identical assessment conditions for both groups using the BlazePod-based tests described below. During the intervention, the EG used BlazePod-based drills for training, whereas the CG trained the same capacities with traditional handball-specific drills without electronic devices.

#### 2.3.1. Focus Reactions Test

**Figure 2:**
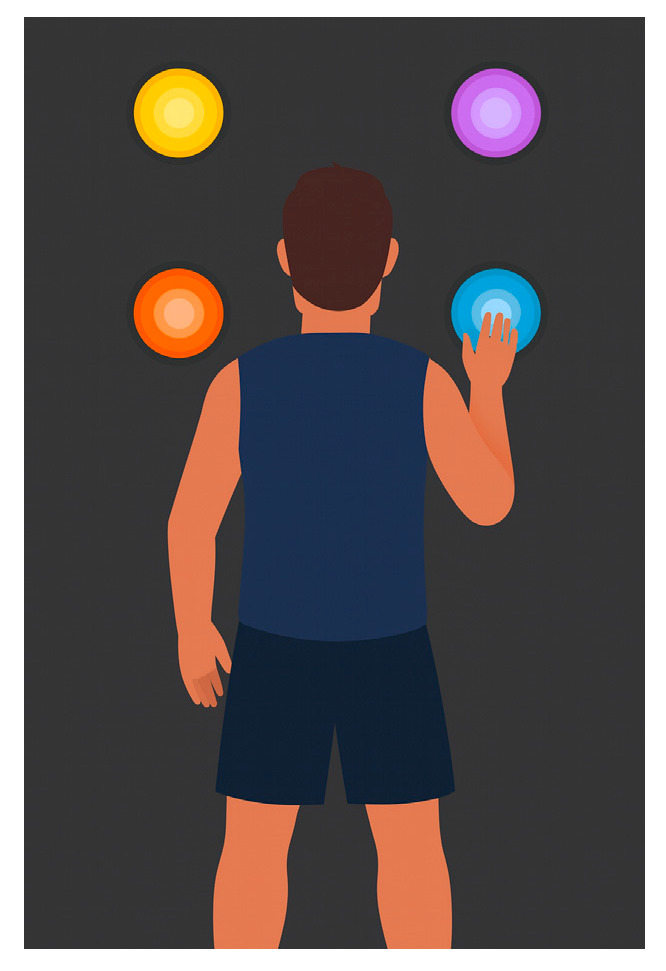
Focus Reactions test.

Four Pods were placed in a square at eye level (Figure 2). The player faced the square and responded manually (right/left) to visual stimuli. At test start, the player had 1.5 s to touch the blue Pod while avoiding other colored Pods (distractors). The 30 s trial assessed reaction time, decision-making, and neural priming. The best score within the window was recorded.

#### 2.3.2. Fast Feet Test

**Figure 3:**
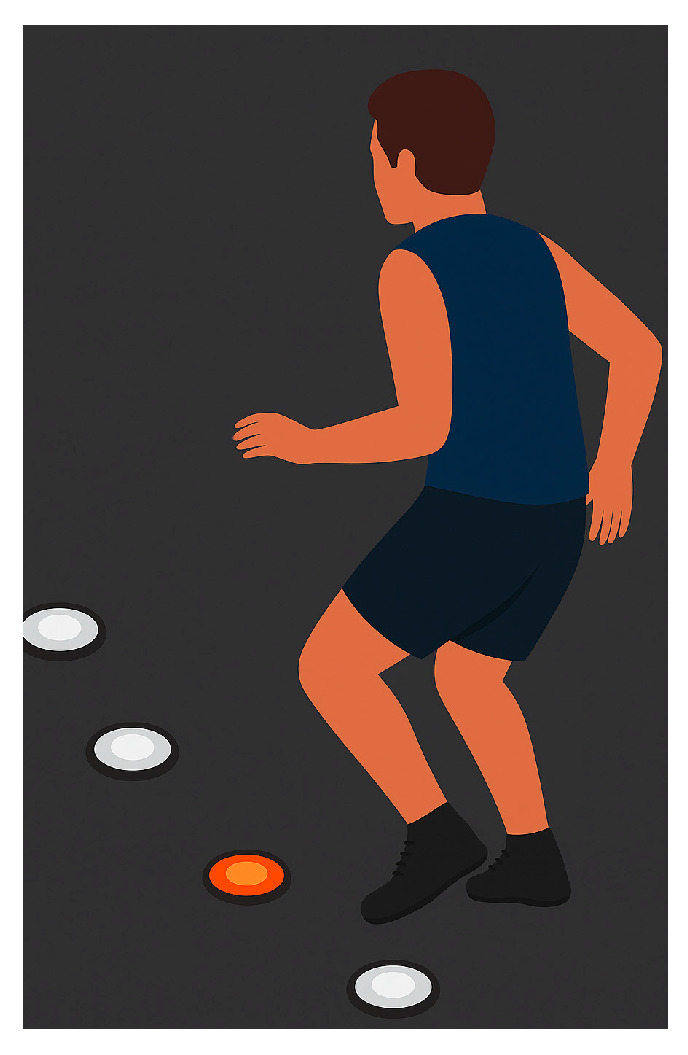
Fast Feet Test.

Four Pods were placed in a line on the floor, 30 cm apart. The player started 20 cm in front (Figure 3). When Pods lit up, the athlete moved quickly to touch the lit Pod nearest to the foot. Pods switched off every 2 s, requiring fast responses. The 30 s test assessed brain activity, reaction time, and decision-making; the best stage performance was recorded.

#### 2.3.3. Clap Challenge Test

**Figure 4:**
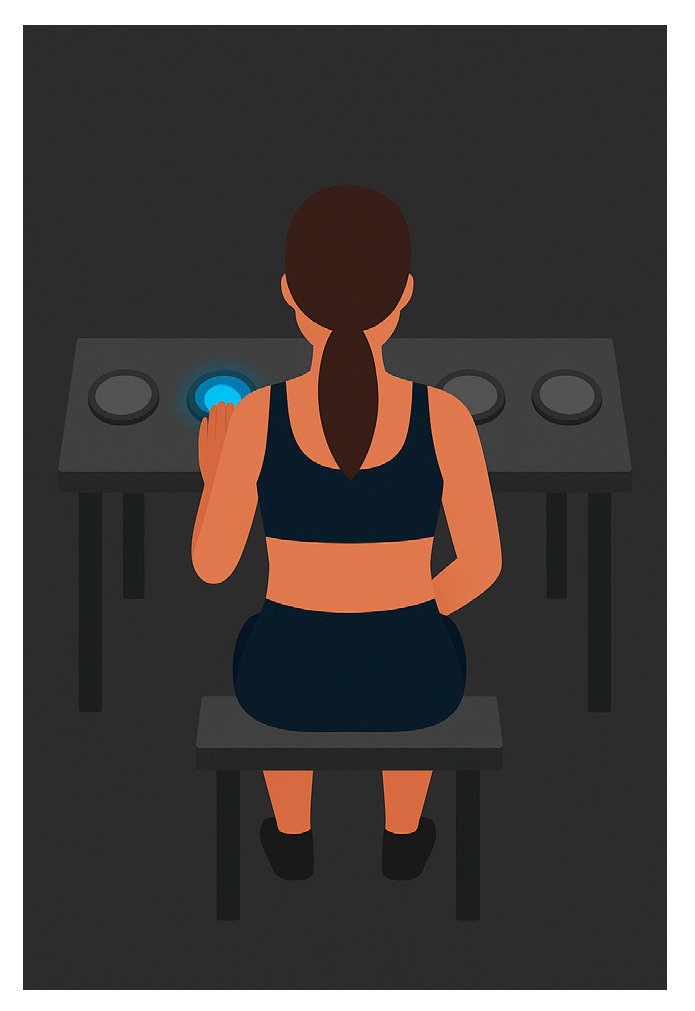
Clap Challenge Test.

Four Pods were placed in a line on a table, 20 cm apart; the athlete sat in front of an 80 cm high table (Figure 4). The athlete touched the lit Pod as often as possible with palm or fingers (right/left) for 30 s. The aim was to assess hand–eye coordination and reaction speed; the best performance was recorded.

#### 2.3.4. Agility T Test

**Figure 5:**
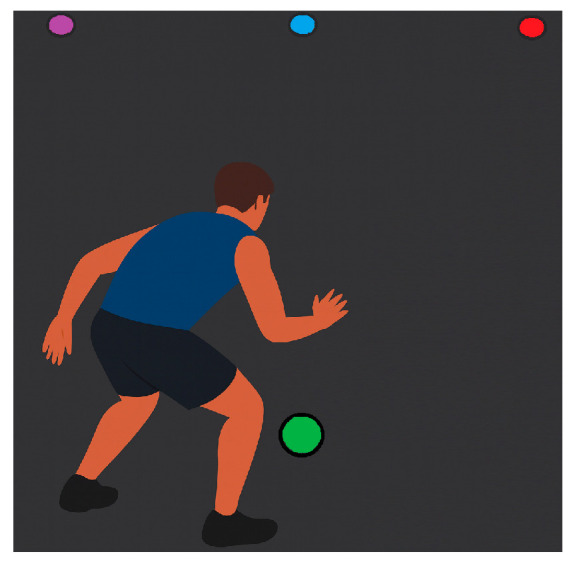
Agility T Test.

Four Pods formed a “T”. The horizontal line measured 10 m; the central blue Pod was 5 m from the purple (left) and red (right) Pods (Figure 5). The vertical distance from the green starting Pod to the blue center Pod was 10 m. From the start, the athlete ran to the blue center Pod, shuffled right to the red Pod, shuffled left to the purple Pod, returned to the blue Pod, then ran backwards to the green starting Pod. Athletes faced forward and avoided crossing legs during lateral moves. Because the movement sequence was pre-planned and not externally stimulus-driven, the Agility T Test was interpreted as a measure of change-of-direction (COD) performance and movement quickness, rather than reactive agility in its strict sense.

### 2.4. Training Protocol

The experimental group (EG), consisting of athletes from the “Handball Academy 2021” Cluj-Napoca, participated in this study with the approval of the club’s Board of Directors and with the consent of their parents.

Both groups trained three times per week for eight weeks. Each training session lasted 90 min in both groups. In both the EG and CG, a 30 min block at the beginning of the session was dedicated to neuromotor training targeting visuomotor reaction performance, change-of-direction quickness, and bilateral task execution; this block was workload-matched between groups (work-bout duration, recovery duration, number of repetitions, and total time-on-task).

In the EG, the 30 min neuromotor block replaced the traditional drills normally used to develop these capacities and was delivered using BlazePod-based tasks. Each block followed a standardized internal structure: after a brief explanation of the tasks (approximately 2–3 min), athletes performed a series of visuomotor and change-of-direction drills organized into short work bouts (20–30 s), interspersed with brief recovery periods (20–40 s). Exercises were conducted in small groups to ensure continuous engagement and comparable exposure time for all participants.

In the CG, the same neuromotor capacities were trained within the corresponding 30 min block using traditional handball-specific exercises without electronic devices (e.g., coach-delivered visual/aural cues, cone- and ball-based coordination drills, and planned change-of-direction tasks), with the same bout structure and total workload as the EG.

Across sessions, athletes in both groups completed the same bout structure (number of work bouts, bout duration, recovery duration) and the same total time-on-task, with total training volume kept consistent within and across sessions. Progression of difficulty was implemented across the intervention period by systematically modifying task constraints rather than increasing overall duration or volume. In the EG, progression was achieved by adjusting stimulus frequency, spatial arrangement of the pods, response constraints (e.g., left/right selection, bilateral execution), and movement demands (e.g., increased range of motion or number of direction changes). In the CG, progression was achieved by analogous modifications of task constraints (e.g., cue frequency, spatial layout, response rules, and change-of-direction demands) within traditional drills.

During the intervention period, tasks were systematically varied in both groups so that athletes practiced a range of visuomotor and change-of-direction demands rather than repeatedly performing the exact assessment conditions.

### 2.5. Statistical Analysis

Data were analyzed using MedCalc^®^ Statistical Software version 23.1.6 (MedCalc Software Ltd., Ostend, Belgium) [51]. Descriptive statistics (mean ± SD) were computed for all variables. The Shapiro–Wilk test was applied to verify the normality of data distributions. Paired-samples *t*-tests were used to assess within-group (pre–post) differences, while independent-samples *t*-tests were performed to compare between-group (experimental vs. control) differences at each time point. Statistical significance was set at *p* < 0.05 (two-tailed). Effect sizes were calculated using Cohen’s d, interpreted as small (0.20), medium (0.50), or large (0.80).

Effect sizes were calculated using Cohen’s d and were interpreted according to conventional thresholds. Given the relatively small sample size and the low within-group variability observed for some outcomes, larger effect size estimates may occur and should be interpreted with caution in conjunction with absolute changes in mean values.

The study followed the Declaration of Helsinki and the ethical guidelines for human-subject research. The study was approved by the Institutional Ethics Committee of Pegaso Telematic University (PROT/E 002466, 29 March 2024) prior to data collection. All procedures adhered to institutional ethical standards and national regulations concerning the protection of study participants.

## 3. Results

**Participant Characteristics.** The experimental group (EG) had an average age of 13.37 years, an average height of 176.25 cm, and an average body mass of 62.99 kg. The control group (CG) had an average age of 13.06 years, a height of 166.12 cm, and a body mass of 61.58 kg (Table 1).

**Within-Group Effects—Control Group (CG).** A significant change was observed only for Focus_Reactions, which increased from M1 (M = 680.06, SD = 75.70) to M2 (M = 689.94, SD = 84.41), *p* = 0.016, *d* = 0.20, indicating slower responses (higher values = worse). All other variables did not change significantly between M1 and M2 (Table 2).

**Within-Group Effects—Experimental Group (EG).**

Two variables improved significantly:

✓ Focus_Reactions decreased from M1 (M = 709.50, SD = 51.00) to M2 (M = 638.31, SD = 66.23), *p* = 0.002, d = −1.20, indicating faster responses.
✓ AgilTT_ShuffleLeft completion time decreased from M1 (M = 4.06, SD = 1.00) to M2 (M = 3.39, SD = 0.23), *p* = 0.014, d = −0.90, reflecting improved agility.

No other variables showed significant pre–post changes (Table 3).

**Inter-Group Comparison at Pre-Test (M1).**

No significant differences were found between the EG and CG for any variable, indicating baseline homogeneity (Table 4).

**Inter-Group Comparison at Post-Test (M2).**

At M2, no significant differences were found for Focus_Reactions (*p* = 0.064), AgilTT_ShuffleRight_B (*p* = 0.186), and AgilTT_SprintOut (*p* = 0.157). Significant between-group differences favored the EG for Fast_Feet (*p* = 0.036), Clap_Challenge (*p* = 0.008), AgilTT_Overall (*p* < 0.001), AgilTT_SprintBack (*p* = 0.003), AgilTT_ShuffleRight_A (*p* = 0.006), and AgilTT_ShuffleLeft (*p* = 0.009) (Table 5).

### 3.1. Focus_Reactions Variable

**Figure 6:**
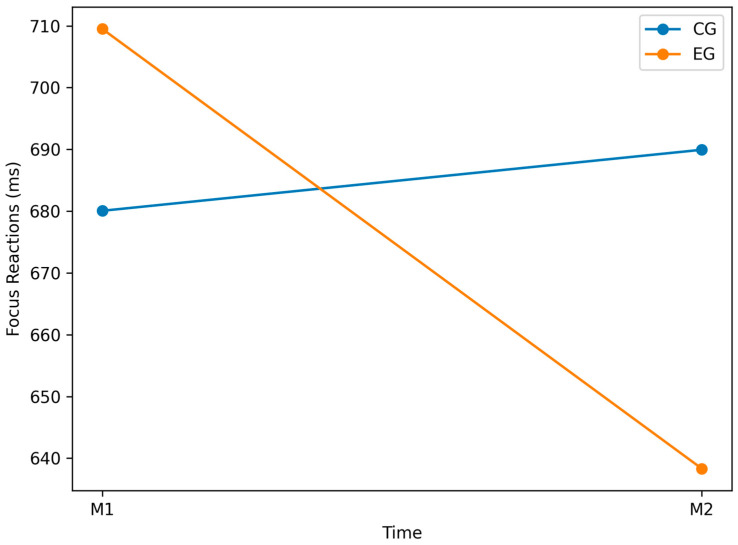
Graphical representation of Focus_Reactions for EG vs. CG at M1 and M2 (lower values = faster reactions).

In the CG, reaction times increased from M1 (M = 680.06, SD = 75.70) to M2 (M = 689.94, SD = 84.41), *p* = 0.016 (Table 2), indicating slower responses. In the EG, reaction times decreased from M1 (M = 709.50, SD = 51.00) to M2 (M = 638.31, SD = 66.23), *p* = 0.002 (Table 3), indicating faster responses. No between-group difference was present at baseline (*p* = 0.207; Table 4). At M2, the between-group difference approached significance (*p* = 0.064; Table 5). Figure 6 illustrates pre–post trends in both groups.

This enhancement may be attributed to the intervention, greater familiarity with the testing procedure, or other contributing factors. When comparing groups (inter-group analysis), no statistically significant difference was observed at baseline (M1; *p* = 0.207), indicating similar initial performance levels (Table 4). At post-test (M2), the difference approached but did not reach statistical significance (*p* = 0.064, d = −0.68; Table 5). Overall, these results suggest that while the EG improved markedly and the CG experienced a slight decline, the intervention’s effect was not strong enough to produce a statistically significant between-group difference at M2. The observed trends should be interpreted cautiously, as changes could be influenced by extrinsic factors such as fluctuations in motivation, fatigue, or learning effects. Nevertheless, the opposite directions of change in the CG and EG may indicate differing responses to the experimental conditions, warranting further investigation to clarify the mechanisms behind these outcomes.

### 3.2. Fast_Feet Variable

No significant within-group changes were observed in the CG (*p* = 0.164; Table 2) or EG (*p* = 0.620; Table 3). Baseline performance did not differ between groups (*p* = 0.078; Table 4). At post-test (M2), a significant between-group difference favored the EG (*p* = 0.036; Table 5).

**Figure 7:**
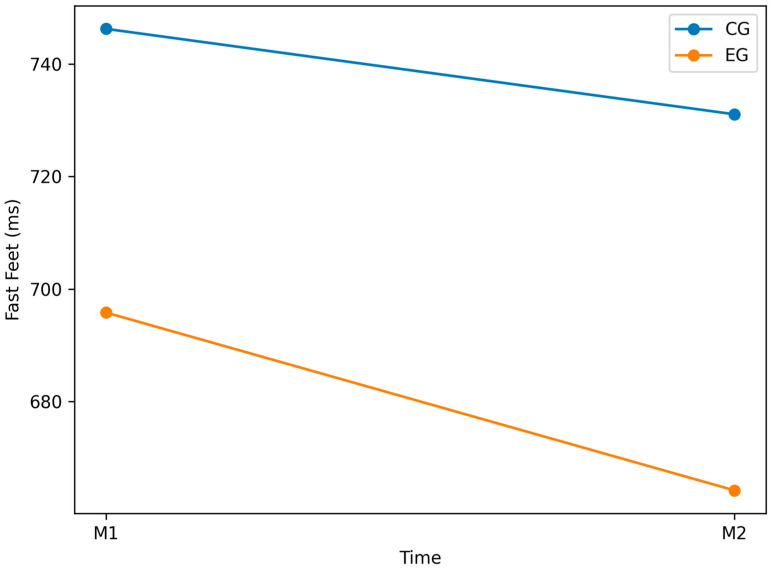
Graphical representation of the Fast Feet variable (EG vs. CG) at pre-test (M1) and post-test (M2). Lower scores indicate better performance.

Figure 7 shows the comparison between groups over time. These findings indicate that, despite the absence of significant within-group changes, the experimental protocol was associated with superior lower-limb movement quickness and coordination at post-test compared to standard training.

The inter-group comparison confirmed comparable baseline performance at M1 (*p* = 0.078, d = 0.65; Table 4). However, at M2, a statistically significant difference emerged between the EG and CG (*p* = 0.036, d = −0.77; Table 5), indicating that the experimental protocol elicited measurable improvements relative to the control condition. Overall, although no intra-group improvements were detected, the significant between-group difference at M2 suggests that the intervention may have had a positive effect on lower-limb movement quickness and coordination. Nonetheless, the magnitude of this effect appears insufficient to produce consistent changes within the EG alone. Future research could explore more targeted neuromotor training elements and longer intervention periods to better evaluate improvements in Fast_Feet performance.

### 3.3. Clap_Challenge Variable

**Figure 8:**
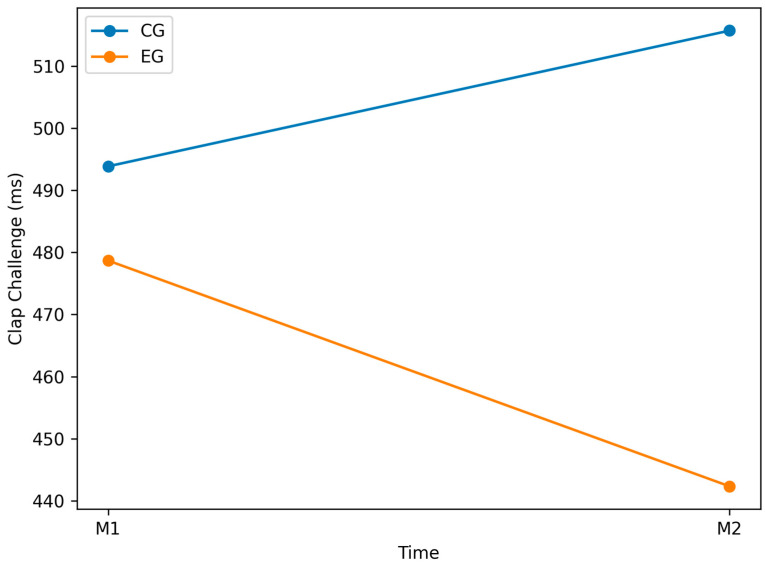
Graphical representation of the Clap Challenge variable for both groups (EG vs. CG) at pre-test (M1) and post-test (M2). Lower values indicate faster reaction times and better performance.

No significant within-group differences were observed in either the CG (*p* = 0.597; Table 2) or EG (*p* = 0.400; Table 3). Baseline performance was comparable between groups (*p* = 0.636; Table 4). At post-test (M2), however, the EG outperformed the CG, showing significantly faster reaction times (*p* = 0.008; Table 5). Figure 8 illustrates the differences between groups across the two testing sessions. These findings indicate that the experimental training protocol was associated with superior hand–eye coordination and reaction speed at post-test compared to standard training, despite the absence of statistically significant within-group effects.

Similarly, in the EG, mean performance improved from M1 (M = 478.69, SD = 94.11) to M2 (M = 442.31, SD = 44.17), reflecting a numerical reduction in reaction time; however, this change was not statistically significant (*p* = 0.400; Table 3).

Inter-group analysis confirmed comparable baseline performance at M1 (*p* = 0.636; Table 4). At post-test (M2), the EG outperformed the CG (*p* = 0.008; Table 5), indicating that the BlazePod-based training enhanced reaction speed and hand–eye coordination compared to standard training.

Although intra-group effects in the EG were not significant, the significant between-group difference suggests a measurable benefit of the intervention. This likely reflects the specific neuromotor demands of the BlazePod protocol. Future studies should extend the intervention duration to consolidate these gains and minimize intra-group variability.

### 3.4. AgilTT_Overall (s) Variable

**Figure 9:**
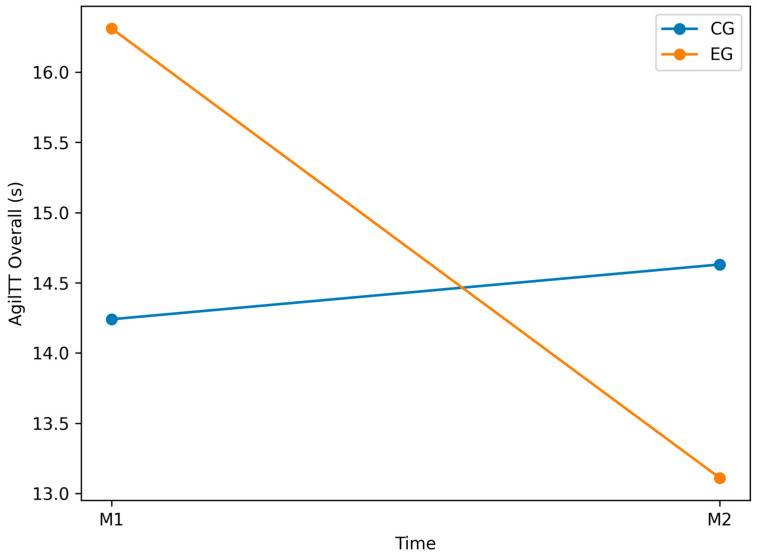
Graphical representation of the Agility T Test variable (test time, s) for both groups (EG vs. CG) at pre-test (M1) and post-test (M2). Lower values indicate better agility performance.

The AgilTT_Overall variable, which represents the total time required to complete the agility course, showed no statistically significant within-group differences in either the CG or EG. However, post-test comparisons revealed a significant between-group effect in favor of the EG, suggesting greater overall agility improvements following BlazePod-based training (Figure 9).

In the CG, mean scores increased from M1 (M = 14.24, SD = 1.43) to M2 (M = 14.63, SD = 1.12), suggesting a slight decline in agility performance, although this change was not statistically significant (*p* = 0.177; Table 2). This indicates that standard training did not produce measurable gains in overall agility.

In contrast, the EG showed a numerical improvement, with mean scores decreasing from M1 (M = 16.31, SD = 7.98) to M2 (M = 13.11, SD = 0.82); however, this change did not reach statistical significance (*p* = 0.089, *d* = −0.6; Table 3), likely due to the high inter-individual variability and relatively small sample size. In this context, the between-group comparison provides a more informative estimate of training-related effects, as it accounts for baseline variability and contrasts performance changes relative to a control condition.

Baseline inter-group analysis revealed no significant difference (*p* = 0.317; Table 4), confirming comparable initial performance. At post-test (M2), however, the EG outperformed the CG (*p* < 0.001; Table 5), indicating that BlazePod-based training was more effective than standard training in enhancing agility.

Although within-group effects in the EG did not reach statistical significance, the clear between-group difference supports the intervention’s efficacy. The absence of within-group significance may be attributed to baseline variability, which reduced statistical power. Future studies should increase sample size, extend intervention duration, and incorporate targeted agility drills to better capture individual performance gains.

### 3.5. AgilTT_SprintBack (s) Variable

The AgilTT_SprintBack variable, which measures the time required to complete the backward sprint segment of the agility test, showed no statistically significant within-group changes for the control group (CG). Mean scores increased from M1 (M = 3.47, SD = 0.48) to M2 (M = 3.58, SD = 0.52), but this change was not significant (*p* = 0.332; Table 2), indicating no measurable improvement in backward sprint performance under standard training conditions.

In the experimental group (EG), mean scores decreased from M1 (M = 3.51, SD = 0.90) to M2 (M = 3.12, SD = 0.23), reflecting improved performance. Although this result approached the conventional threshold for statistical significance, it did not reach it (*p* = 0.090, *d* = −0.6; Table 3), suggesting that the intervention may have had a positive effect but that inter-individual variability limited the ability to detect a consistent within-group change.

At baseline (M1), no statistically significant differences were found between the EG and CG (*p* = 0.847; Table 4), confirming comparable initial performance. At post-test (M2), however, the inter-group comparison revealed a statistically significant difference in favor of the EG (*p* = 0.003; Table 5), indicating that the BlazePod-enhanced training program produced superior improvements in AgilTT_SprintBack performance compared to standard handball training.

Overall, while the within-group change in the EG did not reach statistical significance, the between-group results suggest that the intervention was associated with superior backward sprint performance compared to standard training. The inclusion of drills emphasizing rapid reaction and directional change likely contributed to these gains, underscoring the value of between-group analyses for detecting training effects when high inter-individual variability may obscure within-group outcomes.

### 3.6. AgilTT_ShuffleRight_A (s) Variable

The AgilTT_ShuffleRight_A variable, which assesses lateral agility through rightward shuffling, showed no significant within-group changes in the CG. Mean scores slightly decreased from M1 (M = 2.19, SD = 0.58) to M2 (M = 2.10, SD = 0.19), but this difference was not significant (*p* = 0.170; Table 2), indicating that standard training did not improve rightward lateral movement.

In the EG, mean scores also decreased from M1 (M = 2.49, SD = 1.85) to M2 (M = 1.92, SD = 0.14), reflecting a numerical improvement that did not reach statistical significance (*p* = 0.320; Table 3), likely due to high intra-group variability or limited test sensitivity.

At baseline, no significant difference was found between groups (*p* = 0.533; Table 4), confirming comparable initial performance. At post-test (M2), the EG outperformed the CG (*p* = 0.006; Table 5), indicating that BlazePod-based training enhanced rightward lateral agility compared with standard practice.

Although within-group effects were not statistically significant, the clear between-group difference supports an association between the intervention and superior neuromotor performance in rightward lateral movement compared to standard training. These findings highlight the importance of incorporating rightward agility drills in neuromotor training programs and demonstrate the utility of between-group analyses when high intra-individual variability may mask individual effects.

### 3.7. AgilTT_ShuffleLeft (s) Variable

The AgilTT_ShuffleLeft variable, which evaluates lateral agility through leftward shuffling, showed no significant within-group changes in the CG. Mean scores increased slightly from M1 (M = 3.68, SD = 0.46) to M2 (M = 3.84, SD = 0.60), but this difference was not significant (*p* = 0.107; Table 2), indicating that standard training did not improve leftward agility.

In contrast, the EG demonstrated a statistically significant reduction in mean scores from M1 (M = 4.06, SD = 1.00) to M2 (M = 3.39, SD = 0.23), reflecting a clear improvement in leftward lateral agility (*p* = 0.014; Table 3).

Inter-group analysis confirmed comparable baseline performance (*p* = 0.183; Table 4), while post-test results showed a significant difference in favor of the EG (*p* = 0.009; Table 5).

These findings indicate that AgilTT_ShuffleLeft was among the most responsive variables to the BlazePod-based training program. The improvements observed both within and between groups suggest a consistent and robust effect of the intervention on leftward agility. The drills likely enhanced neuromotor components such as reaction time, coordination, and directional speed, all contributing to the observed gains. These results support the inclusion of lateral agility tasks in youth training and indicate that BlazePod-assisted protocols are associated with meaningful improvements in leftward lateral agility performance.

### 3.8. AgilTT_ShuffleRight_B (s) Variable

The AgilTT_ShuffleRight_B variable, which assesses rightward lateral movement performance in a variant of the agility task, showed no statistically significant within-group changes in either study group. In the control group (CG), mean scores decreased slightly from M1 (M = 2.19, SD = 0.43) to M2 (M = 2.16, SD = 0.44), but the change was not significant (*p* = 0.276; Table 2), indicating that standard training did not elicit measurable improvements in this specific component of lateral agility.

In the experimental group (EG), mean scores improved from M1 (M = 3.33, SD = 1.86) to M2 (M = 2.00, SD = 0.17), but this reduction was also not statistically significant (*p* = 0.299; Table 3). The high baseline variability likely reduced the statistical power to detect intra-group effects despite the apparent numerical improvement.

Inter-group comparisons revealed no significant differences at baseline (M1; *p* = 0.359; Table 4), confirming equivalent initial performance. At post-test (M2), the difference remained non-significant (*p* = 0.186; Table 5), suggesting that the intervention did not yield a detectable advantage for the EG over the CG in this task.

Unlike other agility-related measures, such as AgilTT_ShuffleLeft or AgilTT_ShuffleRight_A, which demonstrated significant post-intervention gains, AgilTT_ShuffleRight_B did not exhibit a measurable training effect. This discrepancy may reflect high inter-individual variability, limited test sensitivity, or insufficient targeting of the specific motor patterns involved in this version of the shuffle task. Future interventions may benefit from incorporating more repetitions, refined drill designs, and greater specificity in rightward lateral movement training to better address the neuromotor demands of AgilTT_ShuffleRight_B.

### 3.9. AgilTT_SprintOut (s) Variable

The AgilTT_SprintOut variable, which evaluates linear sprinting ability within the agility test, showed no statistically significant within-group changes in either study group. In the control group (CG), mean scores increased from M1 (M = 2.68, SD = 0.47) to M2 (M = 2.95, SD = 0.49), but this change was not significant (*p* = 0.920; Table 2), indicating that standard training did not improve sprint performance in this segment of the test.

In the experimental group (EG), mean scores decreased from M1 (M = 2.90, SD = 1.29) to M2 (M = 2.67, SD = 0.59), reflecting a numerical improvement in linear sprint performance; however, this reduction was not statistically significant (*p* = 0.251; Table 3).

Inter-group comparisons confirmed no significant differences at baseline (M1; *p* = 0.527; Table 4), indicating equivalent starting performance. At post-test (M2), the difference also remained non-significant (*p* = 0.157; Table 5), showing that the intervention did not lead to a measurable advantage for the EG over the CG in AgilTT_SprintOut performance.

These results suggest that the BlazePod-based training protocol did not substantially influence linear sprint ability within the study period. It is possible that the intervention primarily targeted lateral agility, coordination, and reaction time rather than straight-line acceleration. Additionally, the high inter-individual variability observed—particularly in the EG at M1—may have reduced the statistical power to detect potential effects.

Future programs aiming to improve AgilTT_SprintOut performance should incorporate sprint-specific drills, such as resisted sprints, high-intensity intervals, or technique-focused exercises, and consider extending session duration or frequency to better stimulate linear speed adaptations.

## 4. Discussion

This study examined the effects of a structured BlazePod-based training program on coordination, movement quickness, and reaction time in adolescent handball players.

Importantly, training frequency, session duration, and the workload of the neuromotor block were matched between groups, indicating that the observed between-group differences are attributable to the training modality (BlazePod-based vs. traditional drills) rather than to differences in training dose.

The findings indicate that targeted, technology-assisted training can effectively enhance neuromotor performance, particularly in reaction time and lateral agility—key components of handball performance. Although not all variables demonstrated statistically significant within-group improvements, the Experimental Group (EG) showed more consistent and pronounced enhancements than the Control Group (CG) across multiple performance domains.

Notably, AgilTT_ShuffleLeft exhibited a significant improvement both within the EG and compared to the CG, suggesting that leftward lateral agility is particularly responsive to this type of intervention. Moreover, inter-group analyses revealed significant advantages for the EG in Fast_Feet, Clap_Challenge, AgilTT_Overall, AgilTT_SprintBack, and AgilTT_ShuffleRight_A, confirming the efficacy of the program in improving both upper- and lower-limb coordination and reaction speed.

These findings align with previous studies that examined various reaction types across different skill levels [52]. Prior research has also explored the variables influencing human reaction time and the relationship between physical activity and its development potential in different sports [14]. Reaction time is influenced by both sensory and motor processes and serves as a reliable indicator of cognitive–motor integration [21,26,53]. By combining visual stimuli with rapid motor responses, the light-based training system likely promoted neural adaptation and enhanced motor execution, consistent with evidence supporting the integration of technological tools into athletic training [14,26].

From a biomechanical perspective, the most responsive variables in this study were those involving lateral agility, which require rapid deceleration, directional changes, and re-acceleration—movements that are highly relevant to handball match situations. The structured use of light-based stimuli may have provided sport-specific neuromotor challenges not adequately addressed by standard training, thereby explaining the EG’s superior performance in agility-based parameters.

The absence of significant within-group improvements in variables such as AgilTT_SprintOut and AgilTT_ShuffleRight_B may be attributed to high inter-individual variability, relatively short intervention duration, or the insufficient targeting of specific motor patterns. Future studies should consider extending the training period, increasing sample size, and including more task-specific drills to optimize performance gains in these less responsive variables.

The present findings also contribute to the growing body of knowledge on how age, sex, and sport-specific demands interact with neuromotor development, highlighting how intelligent, targeted efforts can optimize preparation and enhance success more efficiently than traditional methods [26,52,54]. Because reaction time integrates both sensory and motor systems and reflects processing speed, it represents an excellent index of cognitive-level functioning [55]. Furthermore, our results are consistent with studies investigating reaction time development across age and gender [54], reinforcing the importance of age-appropriate neuromotor stimulation in youth athletic training.

In practical terms, this study suggests that integrating cognitive–motor drills using interactive technologies can enhance sport-specific performance while simultaneously stimulating cognitive processing speed. Coaches and educators could strategically incorporate such protocols into handball training sessions to improve agility, reaction time, and coordination, providing a time-efficient and engaging approach to performance enhancement in young athletes.

Despite the positive findings, some limitations related to the use of the BlazePod system [56] should be acknowledged. Light-based reaction technologies primarily provide controlled and simplified stimulus–response conditions, which may only partially reflect the perceptual and decision-making demands encountered during real match situations. Consequently, ecological validity may be limited when results are directly transferred to competitive contexts. In addition, the cost of the equipment and the need for adequate space and supervision may restrict accessibility for some youth sport programs, particularly in non-professional settings.

### 4.1. Limitations

This study has several limitations that should be acknowledged.

First, the relatively small sample size may have reduced statistical power, particularly for variables with high inter-individual variability (e.g., AgilTT_SprintOut, AgilTT_ShuffleRight_B).

Second, the intervention period was relatively short, possibly limiting the extent of neuromotor adaptations.

Third, because both groups repeatedly practiced drills targeting the assessed capacities, part of the observed improvements may reflect task-specific learning and familiarization effects; however, training dose was matched, and the between-group differences suggest an additional effect attributable to the BlazePod-based stimulus–response modality. Therefore, the present findings should be interpreted as gains in trained visuomotor and change-of-direction performance rather than as generalized enhancements of all coordination domains.

Fourth, while the training protocol was standardized, differences in individual engagement, motivation, and adherence may have influenced performance outcomes.

Fifth, biological maturation (pubertal stage) was not controlled, which could have affected performance changes across participants.

Finally, the study focused exclusively on adolescent handball players, limiting the generalizability of findings to other sports or age groups. An additional limitation concerns the interpretation of effect size estimates. In small samples, effect sizes such as Cohen’s d may be inflated when within-group variability is low. Although all calculations were double-checked for consistency across tables, effect sizes should be interpreted alongside raw score changes rather than as standalone indicators of intervention magnitude. Future studies with larger samples may benefit from reporting confidence intervals to provide a more precise estimation of effects.

### 4.2. Practical Implications for Coaches

From a practical perspective, the present findings suggest that technology-assisted neuromotor training can be effectively integrated into regular handball practice to enhance visuomotor reaction performance and change-of-direction abilities in young athletes. Coaches may incorporate short, structured light-based drills at the beginning of training sessions as an alternative modality for the neuromotor/coordination block within regular practice while keeping overall session duration unchanged. Such drills can be particularly useful during developmental stages, as they allow for high engagement, controlled progression of task difficulty, and targeted stimulation of perceptual–motor skills without substantially increasing overall training load.

### 4.3. Future Directions

Future research should aim to recruit larger and more diverse samples, include control for maturational status, and extend the duration of interventions to allow more robust neuromotor adaptations.

Long-term follow-up testing would be valuable to assess retention of improvements over time.

Moreover, incorporating sport-specific match analyses could help determine whether gains in agility and reaction time translate into improved competitive performance.

Finally, combining light-based reaction drills with plyometric or balance-focused exercises may create a more comprehensive and ecologically valid training approach for young athletes.

## 5. Conclusions

In conclusion, the findings of this study demonstrate that the implemented light-based training program produced significant improvements in several motor performance parameters within the Experimental Group (EG), most notably in Focus_Reactions and AgilTT_ShuffleLeft.

Furthermore, post-test (M2) comparisons revealed significant advantages for the EG over the Control Group (CG) in Fast_Feet, Clap_Challenge, AgilTT_Overall, AgilTT_SprintBack, AgilTT_ShuffleRight_A, and AgilTT_ShuffleLeft.

Although not all intra-group changes reached statistical significance, the consistent pattern of between-group differences supports the efficacy of the intervention in enhancing key aspects of neuromotor performance.

These results underscore the potential of integrating technology-assisted agility and reaction training into educational and youth sport contexts.

Extending intervention duration, refining task specificity, and optimizing training intensity could further increase its impact.

Overall, this study supports the adoption of structured, technology-enhanced motor development programs in physical education and youth sport, offering promising directions for future research and practical application.

## Figures and Tables

**Table 1:** Average values for body mass (MS), height (H), and age (V) in the Control Group (CG) and Experimental Group (EG) at pre-test (M1) and post-test (M2).

Group	Variable	Measurement	Average
CG	MS (kg)	M1	61.59
		M2	62.22
	H (cm)	M1	166.13
		M2	166.81
	V (years)	-	13.06
EG	MS (kg)	M1	62.99
		M2	63.41
	H (cm)	M1	176.25
		M2	177.19
	V (years)	-	13.38

Note. CG = Control Group; EG = Experimental Group; MS = body mass; H = height; V = age; M1 = pre-test; M2 = post-test. Units: kilograms (kg), centimeters (cm), and years.

**Table 2:** Within-group comparisons for the Control Group (CG) between pre-test (M1) and post-test (M2).

Variable	Group	Average	Standard Deviation	*p*	Cohen’s d
*Focus_Reactions*	CG_M1	680.06	75.70	0.016	0.2
	CG_M2	689.94	84.41		
*Fast_Feet*	CG_M1	746.25	85.25	0.164	−0.1
	CG_M2	731.06	86.61		
*Clap_Challenge*	CG_M1	493.88	85.34	0.597	0.2
	CG_M2	515.75	93.51		
*AgilTT_Overall* (s)	CG_M1	14.24	1.43	0.177	0.3
	CG_M2	14.63	1.12		
*AgilTT_SprintBack* (s)	CG_M1	3.47	0.48	0.332	0.4
	CG_M2	3.58	0.52		
*AgilTT_ShuffleRight_A* (s)	CG_M1	2.19	0.58	0.170	−0.2
	CG_M2	2.10	0.19		
*AgilTT_ShuffleLeft* (s)	CG_M1	3.68	0.46	0.107	0.4
	CG_M2	3.84	0.60		
*AgilTT_ShuffleRight_B* (s)	CG_M1	2.19	0.43	0.276	−0.1
	CG_M2	2.16	0.44		
*AgilTT_SprintOut* (s)	CG_M1	2.68	0.47	0.920	0.6
	CG_M2	2.95	0.49		

Note. Focus_Reactions, Fast_Feet, and Clap_Challenge are expressed in milliseconds (ms). AgilTT variables are expressed in seconds (s). For time-based variables, lower scores indicate better performance. CG = Control Group; M1 = pre-test; M2 = post-test; *p* = significance level.

**Table 3:** Within-group comparisons for the Experimental Group (EG) between pre-test (M1) and post-test (M2).

Variable	Group	Average	Standard Deviation	*p*	Cohen’s d
*Focus_Reactions*	EG_M1	709.50	51.00	0.002	−1.2
	EG_M2	638.31	66.23		
*Fast_Feet*	EG_M1	695.81	70.16	0.620	−0.5
	EG_M2	664.19	68.72		
*Clap_Challenge*	EG_M1	478.69	94.11	0.400	−0.5
	EG_M2	442.31	44.17		
*AgilTT_Overall* (s)	EG_M1	16.31	7.98	0.089	−0.6
	EG_M2	13.11	0.82		
*AgilTT_SprintBack* (s)	EG_M1	3.51	0.90	0.090	−0.6
	EG_M2	3.12	0.23		
*AgilTT_ShuffleRight_A* (s)	EG_M1	2.49	1.85	0.320	−0.4
	EG_M2	1.92	0.14		
*AgilTT_ShuffleLeft* (s)	EG_M1	4.06	1.00	0.014	−0.9
	EG_M2	3.39	0.23		
*AgilTT_ShuffleRight_B* (s)	EG_M1	3.33	1.86	0.299	−0.4
	EG_M2	2.00	0.17		
*AgilTT_SprintOut* (s)	EG_M1	2.90	1.29	0.251	−0.2
	EG_M2	2.67	0.59		

Note. Focus_Reactions, Fast_Feet, and Clap_Challenge are expressed in milliseconds (ms). AgilTT variables are expressed in seconds (s). For time-based variables, lower scores indicate better performance. EG = Experimental Group; M1 = pre-test; M2 = post-test; *p* = significance level.

**Table 4:** Between-group comparisons (CG vs. EG) at pre-test (M1).

Variable	Group	*t*	df	*p*	Cohen’s d
*Focus_Reactions*	CG_M1	−1.290	30	0.207	−0.46
	EG_M1				
*Fast_Feet*	CG_M1	1.827	30	0.078	0.65
	EG_M1				
*Clap_Challenge*	CG_M1	0.478	30	0.636	−0.17
	EG_M1				
*AgilTT_Overall* (s)	CG_M1	−1.017	30	0.317	0.36
	EG_M1				
*AgilTT_SprintBack* (s)	CG_M1	−0.194	30	0.847	0.07
	EG_M1				
*AgilTT_ShuffleRight_A* (s)	CG_M1	−0.630	30	0.533	0.22
	EG_M1				
*AgilTT_ShuffleLeft* (s)	CG_M1	−1.362	30	0.183	−0.48
	EG_M1				
*AgilTT_ShuffleRight_B* (s)	CG_M1	−0.932	30	0.359	0.33
	EG_M1				
*AgilTT_SprintOut* (s)	CG_M1	−0.640	30	0.527	0.23
	EG_M1				

Note. Independent-samples *t*-tests comparing the Control Group (CG) and Experimental Group (EG) at baseline (M1). Focus_Reactions, Fast_Feet, and Clap_Challenge are expressed in milliseconds (ms). AgilTT variables are expressed in seconds (s). Negative *t*-values indicate that the CG had lower mean scores than the EG. df = degrees of freedom; *p* = two-tailed significance level. For time-based variables, lower scores indicate better performance.

**Table 5:** Between-group comparisons (CG vs. EG) at post-test (M2).

Variable	Group	*t*	df	*p*	Cohen’s d
*Focus_Reactions*	CG_M2	1.925	30	0.064	−0.68
	EG_M2				
*Fast_Feet*	CG_M2	2.189	30	0.036	−0.77
	EG_M2				
*Clap_Challenge*	CG_M2	2.840	30	0.008	−1.0
	EG_M2				
*AgilTT_Overall* (s)	CG_M2	4.378	30	<0.001	−1.55
	EG_M2				
*AgilTT_SprintBack* (s)	CG_M2	3.214	30	0.003	−1.14
	EG_M2				
*AgilTT_ShuffleRight_A* (s)	CG_M2	2.990	30	0.006	−1.06
	EG_M2				
*AgilTT_ShuffleLeft* (s)	CG_M2	2.798	30	0.009	−0.99
	EG_M2				
*AgilTT_ShuffleRight_B* (s)	CG_M2	1.354	30	0.186	−0.48
	EG_M2				
*AgilTT_SprintOut* (s)	CG_M2	1.453	30	0.157	−0.51
	EG_M2				

Note. Independent-samples *t*-tests comparing the Experimental Group (EG) and Control Group (CG) at post-test (M2). Focus_Reactions, Fast_Feet, and Clap_Challenge are expressed in milliseconds (ms). AgilTT variables are expressed in seconds (s). df = degrees of freedom; *p* = two-tailed significance level. For time-based variables, lower scores indicate better performance.

## Data Availability

The data presented in this study are available on request from the corresponding authors.

## Abbreviations

The following abbreviations are used in this manuscript: CG—Control Group; EG—Experimental Group; M1—Measurement 1 (pre-test); M2—Measurement 2 (post-test); M—Mean; SD—Standard Deviation; *p*—*p*-value (Type I error probability); *df*—degrees of freedom; *t*—Student’s *t*-test; s—seconds; AgilTT—Agility T Test; Focus_Reactions—Focus Reactions Test; Fast_Feet—Fast Feet Test; Clap_Challenge—Clap Challenge Test; AgilTT_Overall—Agility T Test total time; AgilTT_SprintBack—Sprint Back segment; AgilTT_ShuffleRight_A—Shuffle Right A segment; AgilTT_ShuffleLeft—Shuffle Left segment; AgilTT_ShuffleRight_B—Shuffle Right B segment; AgilTT_SprintOut—Sprint Out segment.
